# Patient and provider delay in tuberculosis suspects from communities with a high HIV prevalence in South Africa: A cross-sectional study

**DOI:** 10.1186/1471-2334-8-72

**Published:** 2008-05-25

**Authors:** Graeme Meintjes, Hennie Schoeman, Chelsea Morroni, Douglas Wilson, Gary Maartens

**Affiliations:** 1 Division of Infectious Diseases, Department of Medicine, Faculty of Health Sciences, University of Cape Town, Anzio Road, Observatory, 7925, South Africa; 2 GF Jooste Hospital, Duinefontein Road, Manenberg, 7764, South Africa; 3 Women's Health Research Unit, School of Public Health and Family Medicine, Faculty of Health Sciences, University of Cape Town, Anzio Road, Observatory, 7925, South Africa; 4 Division of Clinical Pharmacology, Department of Medicine, Faculty of Health Sciences, University of Cape Town, Anzio Road, Observatory, 7925, South Africa

## Abstract

**Background:**

Delay in the diagnosis of tuberculosis (TB) results in excess morbidity and mortality, particularly among HIV-infected individuals. This study was conducted at a secondary level hospital serving communities with a high HIV prevalence in Cape Town, South Africa. The aim was to describe patient and provider delay in the diagnosis of TB in patients with suspected TB requiring admission, and to determine the risk factors for this delay and the consequences.

**Methods:**

A cross-sectional study was conducted. Patients admitted who were TB suspects were interviewed using a structured questionnaire to assess history of their symptoms and health seeking behaviour. Data regarding TB diagnosis and outcome were obtained from the medical records. Bivariate associations were described using student's T-tests (for means), chi-square tests (for proportions), and Wilcoxon rank-sum tests (for medians). Linear regression models were used for multivariate analysis.

**Results:**

One hundred twenty-five (125) patients were interviewed. In 104 TB was diagnosed and these were included in the analysis. Seventy of 83 (84%) tested were HIV-infected. Provider delay (median = 30 days, interquartile range (IQR) = 10.3–60) was double that of patient delay (median = 14 days, IQR = 7–30). Patients had a median of 3 contacts with formal health care services before referral. Factors independently associated with longer patient delay were male gender, cough and first health care visit being to public sector clinic (compared with private general practitioner). Patient delay ≥ 14 days was associated with increased need for transfer to a TB hospital. Provider delay ≥ 30 days was associated with increased mortality.

**Conclusion:**

Delay in TB diagnosis was more attributable to provider than patient delay, and provider delay was associated with increased mortality. Interventions to expedite TB diagnosis in primary care need to be developed and evaluated in this setting.

## Background

Delay in diagnosis is an important contributor to the excess morbidity and mortality of tuberculosis (TB) in HIV-infected people [1,2]. Delay in the diagnosis of TB in HIV-infected individuals has a greater impact on mortality and morbidity when compared with HIV-uninfected individuals because TB presents more commonly with dissemination [3] and disease progression is more rapid [4]. In addition, HIV disease progression is accelerated and viral load increased by TB [5-7] and a delayed TB diagnosis exacerbates this [8].

There are increased costs associated with a delayed diagnosis, both to the patient in terms of lost employment and visits to the health care system and to the health care system in terms of additional clinic visits and the need for hospitalisation. Also, a delay in diagnosis means that the untreated individual remains an infectious risk in the community for longer, contributing to increased TB transmission [9].

This study quantified diagnostic delay, associated factors, and consequences in patients requiring admission with suspected TB to a community-based secondary level hospital serving communities with a high HIV prevalence in Cape Town, South Africa (GF Jooste Hospital). The hospital serves a population of 1.3 million people. During the study period the main community served by the hospital had an antenatal HIV prevalence of 27% and a TB incidence of 1416/100 000 per annum.

## Methods

This was a cross-sectional study. Data were collected using an interviewer-administered semi-structured questionnaire. All TB suspects who were admitted to the medical wards at GF Jooste Hospital between February and September 2003 were approached while in the ward for participation. Most of the TB diagnostic work-up in this setting takes place at government-funded public sector primary care facilities. Ill patients requiring admission are referred to the hospital by these facilities or by a private general practitioner (GP). The study was conducted prior to antiretroviral therapy (ART) being available in the public sector in South Africa and no patients in the study were on ART. There was also no isoniazid (INH) preventive therapy programme in the public or private sector.

Patients 18 years or older were recruited. Exclusion criteria were treatment for TB within the preceding 6 months, confusion, and being a TB suspect solely on the basis of suspected TB meningitis. Patients were not recruited if admitted over weekends or during the two periods of leave taken by the interviewer. Participants gave written consent. The interviews were conducted by a trained interviewer in the patient's home language. Data on patients' socio-demographic and economic profile, symptoms, timing of seeking medical attention and type of health provider visited, and knowledge, attitudes and beliefs (KAB) were collected. The KAB questions were open-ended and the answers were coded into categories for analysis.

Patients with an unknown HIV serostatus were offered HIV testing with pre- and post- test counselling upon completion of the interview. The test for HIV, using the Abbott Determine HIV-1/2, was conducted in a laboratory. Those with a previous diagnosis of HIV at another institution were not retested as long as this diagnosis was documented in the referral letter. Data regarding TB diagnosis and outcome were obtained from the medical records.

The main inclusion criterion was a TB suspect, defined as referral from a primary care facility for diagnostic work-up for TB or in whom the admitting doctor suspected TB. Patients were followed up to assess whether the final diagnosis made by the admitting doctors was TB, but no attempt was made to influence their diagnostic process. The diagnosis of TB was divided into three categories: possible, probable and definite. The diagnosis was regarded as possible if both the clinical picture and radiological findings were assessed by a senior clinician to be compatible with TB, but microscopy and culture were negative or not done. Probable TB was diagnosed if acid fast bacilli (AFBs) were found on microscopy of any sample, or if granulomas were detected on histological or cytological examination. Definite TB was diagnosed if a culture grew *Mycobacterium tuberculosis *(MTB). Those with a diagnosis of TB were included in analysis.

Patient delay was defined as the time from the first symptom to seeking medical attention in the formal health services. Total delay was defined as time from first symptom to admission to GF Jooste. Provider delay was calculated by subtracting patient delay from total delay. These delays reflected outpatient delay. Inpatient delay was calculated separately, being defined as the duration from the day of admission to the hospital's casualty department to the day TB treatment was initiated. Constitutional symptoms were defined as one or more of tiredness, generalised weakness, anorexia or reported weight loss.

The first author gathered the medical data from patient records. Data were entered on an Epi-info 6 database and statistical analysis was done using STATA 8.0. Frequency distributions and univariate descriptive statistics were used for preliminary analyses. Bivariate associations were described using student's T-tests (for means), chi-square tests (for proportions), and Wilcoxon rank-sum tests (for medians). Variables that demonstrated significant bivariate association (defined as p ≤ 0.05) with patient delay were entered into linear regression models, developed to assess independent effects. Variables were retained in the final model if they demonstrated significant independent association (defined as p ≤ 0.05) with patient delay, or if their removal altered the association between other covariates and patient delay.

Ethical approval for the study was obtained from the University of Cape Town Faculty of Health Sciences Research Ethics Committee (Reference number: 210/2001).

## Results

**Figure 1:**
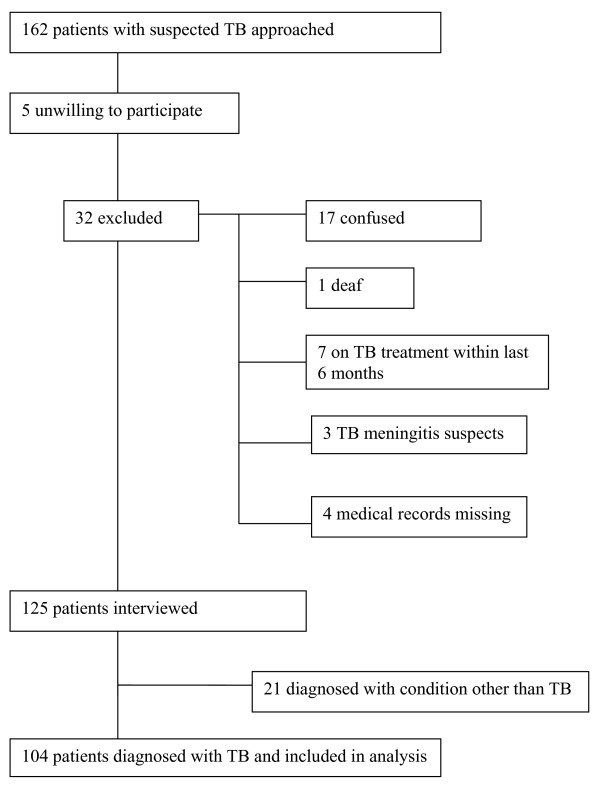
**Reasons for exclusion from study among 162 patients approached**. 162 admitted patients with suspected tuberculosis (TB) were approached to participate in the study, of which 104 are included in the analysis. The reasons for the exclusion of the other patients are shown.

One hundred and sixty two TB suspects were approached for participation. The reasons for exclusion from the study are shown in Figure 1; 125 patients were interviewed.

Among these 125 patients, in 97 a specimen was sent for TB microscopy. TB culture was performed in 62 cases. TB was diagnosed in 83% (n = 104). Among these, TB diagnosis was definite in 39% (n = 41), probable in 33% (n = 34) and possible in 28% (n = 29). TB was pulmonary in 38% (n = 39), pulmonary with extrapulmonary in 56% (n = 58), and extrapulmonary alone in 7% (n = 7). Of these 104 patients, 93 were started on TB treatment, 3 died before TB treatment could be started, and in 3 TB treatment was not started for unclear reasons or because the culture result only came back after discharge. Five patients, in whom the diagnosis of TB was not made during the admission because the diagnosis was not certain, were referred to their local TB clinic for follow up of TB culture results which were subsequently positive. Among those started on TB treatment during the admission the median inpatient delay to starting TB treatment was 1 day. TB treatment was started on the day of admission in 35% (n = 33).

Of the 41 patients who had a culture that grew MTB, 10 had drug susceptibility testing for INH and rifampicin performed using direct proportions method. In one patient INH monoresistance was demonstrated and in another multi-drug resistant MTB. The other 8 demonstrated MTB susceptible to both drugs.

Diagnoses other than TB were made in 17% (n = 21): bacterial pneumonia (n = 9), *Pneumocystis jirovecii *pneumonia (n = 3), chronic lung disease with secondary infection (4, one of whom also had disseminated Kaposi's sarcoma), bacterial empyema (n = 3), lung abscess (n = 1) and bacterial pneumonia and meningitis (n = 1).

Further analysis was restricted to those 104 patients with a diagnosis of TB.

### Patient characteristics

Characteristics of those patients diagnosed with TB are shown in Table 1. Their median age was 30 years (interquartile range, IQR = 25–38.5). Thirty percent (n = 31) had previously had TB, one of whom had defaulted treatment. HIV serostatus was 42% (n = 44) known seropositive before admission, 25% (n = 26) diagnosed seropositive during the study, 13% (n = 13) tested HIV seronegative, and 20% (n = 21) declined HIV testing. HIV infection prevalence was thus 84 % in those in whom status was known (70/83).

**Table 1:** Characteristics of the 104 patients with a diagnosis of tuberculosis

	**n**	**%**
**Gender**		
Male	39	37.5
Female	65	62.5
**First language**		
Xhosa	90	86.5
Afrikaans	6	5.8
Other	8	7.7
**Marital status**		
Single	67	64.4
Married	15	14.4
Living together	18	17.3
Widowed	4	3.8
Dependents*		
Nil	19	18.6
1	26	25.5
2	23	22.5
3	15	14.7
≥4	19	18.6
**Employment status**		
Unemployed	65	62.5
Employed	23	22.1
Grant	10	9.6
Casual work	2	1.9
Student	2	1.9
Pensioner	2	1.9
Formal education**		
None	5	4.9
Primary	22	21.4
Secondary	74	71.8
Studied after school	2	1.9

*Data for 2 patients were missing

**Data for 1 patient were missing

### Symptoms

Their commonest initial symptoms were constitutional (n = 44, 42%), cough (n = 38, 37%) and chest pain (n = 5, 5%). The commonest symptoms to be reported during the course of the illness were: constitutional (n = 95, 91%), cough (n = 81, 78%), febrile symptoms (n = 55, 53%), dyspnoea (n = 52, 50%), chest pain (n = 38, 37%) and gastro-intestinal (n = 31, 30%).

Twenty-five percent (n = 26) were working before they fell ill. Of these, 92% (n = 24) stopped working during their illness. Eighty-four percent (n = 87) were too ill to leave their home for a median of 30 days (interquartile range (IQR) = 21–60) before admission; and 75% (n = 78) had been bed-bound for a median of 10.5 days (IQR = 7–14).

### Health care utilisation

Fifteen percent (n = 16) reported using a home remedy. In a third of these patients, this was a cough mixture. Fourteen percent (n = 15) visited a traditional healer, four of them on more than one occasion. Patients had a median of 3 contacts (IQR = 2–4) with the formal health services at the time of referral to GF Jooste. Eighty-eight percent (n = 91) visited a public sector clinic and 57% (n = 59) visited a GP on one or more occasion during the course of their illness. The health providers visited and patterns of consultation are shown in Table 2.

**Table 2:** Health provider visits prior to admission for the 104 patients with a diagnosis of tuberculosis

	**n**	**%**
**Category of health provider visited**		
Public sector clinic	91	87.5
GP	59	56.7
Traditional healer	15	14.4
Secondary or tertiary hospital	11	10.6
		
**Visited category of provider 3 or more times**		
Public sector clinic	30	28.8
GP	15	14.4
Traditional healer	1	1.0
		
Health provider first visited*		
Public sector clinic	57	55.3
GP	34	33.0
Traditional healer	10	9.7
Secondary or tertiary hospital	2	1.9
		
Provider that referred patient for admission*		
Public sector clinic	70	68.0
GP	24	23.3
Secondary or tertiary hospital	4	3.9
Self-referred	5	4.9
		
**Common patterns of consultation**		
Visited public sector clinic only	34	32.7
Visited public sector clinic and GP	34	32.7
Visited GP only	9	8.7
Visited public sector clinic, GP and traditional healer	8	7.7

GP = Private general practitioner

*Data were missing in one patient for each of these variables

Thirty-eight percent (n = 39) reported having had a sputum specimen taken during the course of their illness before admission and less than one-half of these (n = 18) were told the result of the test (negative in 83%, n = 15). Nine patients were unable to attend for the result because they were too weak. Forty-eight percent (n = 50) reported having had one or more chest radiographs performed prior to admission. GPs requested no sputum samples and 3 chest radiographs in the 59 patients who consulted them.

### Delay

The median total delay from onset of symptoms to admission was 60 days (IQR = 30–90), median patient delay was 14 days (IQR = 7–30) and median provider delay was 30 days (IQR = 10.3–60).

### Factors associated with delay (Table 3)

**Table 3:** Characteristics significantly associated with patient and provider delay in bivariate analysis among the 104 patients with a diagnosis of tuberculosis*

**Patient Delay**		
Characteristic, n (%)	Median Patient Delay (IQR)	p-value**
Gender		
Male, 39 (37.5)	30 (7–60)	0.002
Female, 65 (62.5)	14 (7–21)	
First visit		
Private general practitioner, 34 (33.0)	10.5 (7–14)	0.01
Clinic, 57 (55.3)	14 (7–30)	
Traditional healer, 10 (9.7)	21 (7–60)	
Hospital, 2 (1.9)	30 (30–30)	
Any cough reported		
Yes, 81 (77.9)	14 (7–30)	0.003
No, 23 (22.1)	7 (4–14)	
**Provider Delay**		
Characteristic, n (%)	Median Provider Delay (IQR)	p-value**
Employment status		
Unemployed/grant/pension, 77 (74.0)	39 (15–60)	0.02
Employed/casual work/student, 27 (26.0)	19 (7–46)	

*The following variables were assessed for their bivariate relationship with patient and provider delay: gender, age, marital status, dependents, employment status, educational level, working before illness, type of TB, HIV status, first symptom, symptoms during course of illness, cough as symptom and as first symptom, consulted a traditional healer, place of first visit, sputum in primary care, chest radiograph in primary care, and home remedy used. For those not presented in the table, the associations were found to be non-significant.

**p-values represent comparisons of medians across different categories within each variable.

In bivariate analysis, longer patient delay was associated with male gender, the health care service the patient first visited and cough being reported as a symptom. In multivariate analysis the following factors were independently significantly associated with longer patient delay: male gender (p = 0.02), having cough as a symptom (p = 0.02) and making the first health care visit to a provider other than a GP (p = 0.05).

In bivariate analysis provider delay was associated with employment status. Those who were employed, had casual work or were studying had a shorter delay than those not in employment (19 vs 39 days, p = 0.02). Multivariate analysis was not performed given that only one factor was associated with provider delay in bivariate analysis. Type of TB and HIV status were not associated with patient or provider delay.

### Outcome

Sixty-eight percent (n = 71) were discharged home from GF Jooste (three of whom were known to have died 8, 13 and 14 days after discharge), 11% (n = 11) died in hospital, 20% (n = 21) were referred to a TB hospital and 1 was referred to another hospital. For those who were admitted, the median duration of admission at GF Jooste was 9 days (IQR = 5.5–12).

Patient delay ≥ 14 days compared with delay < 14 days was associated with a significantly increased likelihood of transfer to a TB hospital (17 vs 4%, p = 0.04). Provider delay of ≥ 30 days as compared to < 30 days was significantly associated with mortality (12% vs 2%, p < 0.001).

### Knowledge attitudes and beliefs

Ninety-five percent (n = 99) had heard about TB before their illness. When asked what they knew about TB in an open-ended question, 54% (n = 56) spoke about the treatment, 38% (n = 39) spoke of the cough and/or constitutional symptoms associated with TB and 25% (n = 26) could give no details. 97% (n = 101) had heard about HIV/AIDS before their illness. When asked what they knew about HIV/AIDS, 63% (n = 65) mentioned sexual transmission, 38% (n = 40) mentioned it was a deadly disease or that there is no cure, 13% (n = 14) mentioned the importance of seeking medical attention or joining a support group, 12% (n = 12) mentioned blood transmission, but 28% (n = 29) could give no details. In response to this open-ended question, no patients mentioned that they were aware of a heightened risk of TB in HIV-infected people.

When asked in an open-ended question what they thought was wrong with them when they got sick, 36% (n = 37) said they did not know, 21% (n = 22) said they thought it was HIV (all 22 of these patients were known HIV positive before the admission), 20% (n = 21) thought it was TB, 20% (n = 21) thought they had a cold or flu, 5% (n = 5) attributed it to other medical problems, 3% (n = 3) to bewitchment and 2% (n = 2) to psychological distress.

When patients were asked what made them wait before seeking medical attention, 41% (n = 43) replied that they thought they would get better on their own, 31% (n = 32) replied that they had not waited, 18% (n = 19) that they had competing priorities and 13% (n = 13) cited physical or emotional reasons. Competing priorities cited were a lack of money, having to write exams, having to come to the city for help and having not disclosed HIV status to partner. The physical reasons cited related to being too weak to attend the health services and the emotional reasons related to feeling scared or depressed. When asked what had prompted them to seek medical attention, 56% (n = 58) cited symptoms of weakness or dizziness, 50% (n = 52) shortness of breath, 16% (n = 17) a cough, 13% (n = 14) chest pain, 14% (n = 15) abdominal symptoms, and 5% (n = 5) haemoptysis.

## Discussion

Among those diagnosed with TB provider delay (median 30 days) was double that of patient delay (14 days). This finding suggests that important diagnostic delays are encountered in the primary care services by patients seeking attention for TB symptoms. Patients visited multiple primary health care providers and this lack of continuity in care (or "doctor shopping") likely contributed to the failure to investigate appropriately and provider delay in diagnosis. Other studies have compared patient and provider delay, with some finding diagnostic delay more attributable to patient delay [10-13], but others finding it more related to provider delay [14-18].

The causes for patient delay in our study do not appear to be due to lack of knowledge of TB or HIV (95% or more had heard of TB and HIV). However, the majority of patients were not attributing their symptoms to TB or HIV suggesting that their knowledge of these diseases was not being personalised. In other studies, health knowledge and perceptions, health seeking behaviour and visits to traditional healers have been shown to play a role in delaying diagnosis [10,19-22]. In our study 14% had visited a traditional healer.

Reduced diagnostic sensitivity of sputum smears [23,24] and chest radiographs [25] in HIV-infected people have been reported as reasons for delay in the diagnosis of TB. These factors may have played a role here (in 83 % of those who were told the result of their sputum smear it was negative), but only 38% of patients reported having had a sputum sample sent and 48% a radiograph performed. This suggests that the investigation of patients presenting with TB symptoms to primary care providers could be improved.

Patient delay was found to be longer for males, those who first visited a provider other than a GP and those who had a cough. In contrast, several other studies have demonstrated that longer delay is associated with female gender [13,14,22,26,27], attributed to gender differences in health-seeking behaviour and health provider attitudes and behaviour. Our findings that visiting a GP first was associated with a shorter patient delay suggests that the GP may be more accessible than other providers and that those that can afford GP private fees will visit the GP earlier. The finding that those with cough delay longer could result from people seeking help when their cough has been present for two weeks or longer as suggested by TB public health messages and that in contrast other symptoms such as chest pain and neurological symptoms led them to access the health care system earlier. Cough as the only symptom was also associated with longer delay in another study [16].

Patients required a median of 9 days hospitalisation at GF Jooste and 20% of patients were referred to a TB hospital. Longer patient delay resulted in increased need for referral for inpatient TB treatment. This demonstrates the financial burden carried by the health service as a result of patient delays. More importantly provider delay resulted in increased mortality underlining the importance of interventions to address this. Few other studies have specifically analysed the consequences of diagnostic delay. One study conducted in the Gambia showed that longer total delay was associated with an increased risk of death [28].

Participants in this study were so sick that they required hospitalisation for investigation and treatment, the majority (75%) being bedbound by the time of admission. The fact that patients were so ill on admission after having been ill for a median of 60 days suggests rapid progression of TB. This is probably attributable to the fact that the majority were HIV-infected and the progression of TB is more rapid in HIV-infected people [4]. Hospitalisation is likely to be a frequent consequence of diagnostic delay in HIV-infected people.

There are several limitations of our study. Firstly, we relied upon patient recall of the history of their illness and health seeking pattern, rather than documented records. Because of the fear of criticism that may accompany reporting of a visit to a traditional healer many patients may not have disclosed this. The study was based on patient interviews and thus could not adequately explore health system issues such as referral pathways and investigation practices. A further limitation was that this study was conducted at a secondary hospital and thus only patients who were sick enough to require referral for admission were included. Thereby we focused on those patients who had presented to the health service with TB symptoms, but had not been initiated on TB treatment as outpatients. This subgroup of patients is likely to be at highest risk for a poor outcome as a consequence of delay. Those patients who were timeously investigated and diagnosed with TB at a primary care level are not represented in this study. This does mean that these results are not necessarily generalisable to all patients presenting to the health services with TB symptoms.

## Conclusion

Provider delay was double that of patient delay. Independent risk factors for longer patient delay were male gender, cough and first health care visit being to public sector clinic (compared with a private GP). Patient delay of 14 days or more was associated with increased need for transfer to a TB hospital. Provider delay of 30 days or more was associated with higher mortality.

These findings suggest a need for interventions to expedite the diagnosis of TB in this setting, particularly in HIV-infected patients. Strategies that need to be considered relate to the health system (for example, steps by the public sector to actively involve private GPs in the TB diagnostic process given that the majority of patients visited a GP during their illness), patient health seeking behaviour (for example, education to promote continuity in the providers they visit), alternative diagnostic modalities (for example, the implementation of clinical algorithms for diagnosing smear negative TB) and the need to ensure patients are properly investigated for chronic cough at the primary level and referred to TB clinics.

## Competing interests

The authors declare that they have no competing interests.

## Authors' contributions

GrM conceived of the study, participated in the design, was responsible for data acquisition, participated in data interpretation and drafted the manuscript. HS participated in the design, performed statistical analysis and assisted with interpretation of data. CM performed statistical analysis, assisted with interpretation of data and helped draft the manuscript. DW participated in the design and interpretation of data. GaM conceived of the study and participated in the design, interpretation of data and drafting the manuscript. All authors read and approved the final manuscript.

## Pre-publication history

The pre-publication history for this paper can be accessed here:


